# Evaluation of Fungal Sensitivity to Biosynthesized Copper-Oxide Nanoparticles (CuONPs) in Grapevine Tissues and Fruits

**DOI:** 10.3390/jof11100719

**Published:** 2025-10-06

**Authors:** Domingo Martínez-Soto, Erisneida Campos-Jiménez, Alejandro Cabello-Pasini, Luis Enrique Garcia-Marin, Anaid Meza-Villezcas, Ernestina Castro-Longoria

**Affiliations:** 1 Department of Microbiology, Center for Scientific Research and Higher Education of Ensenada (CICESE), Carretera Tijuana-Ensenada 3918, Zona Playitas, Ensenada 22860, Baja California, Mexico; dmartinez@cicese.edu.mx (D.M.-S.); ecampos@cicese.edu.mx (E.C.-J.); ennriquemarin@gmail.com (L.E.G.-M.); 2 Marine Botany Research Group, Institute of Oceanological Research (IIO), Universidad Autónoma de Baja California (UABC), Ensenada 22860, Baja California, Mexico; acabello@uabc.edu.mx; 3 Center for Nanosciences and Nanotechnology, National Autonomous University of Mexico, Ensenada 22860, Baja California, Mexico; pa_anaid@ens.cnyn.unam.mx

**Keywords:** copper-oxide nanoparticles, control of phytopathogens, *Alternaria alternata*, *Aspergillus niger*, grape plants

## Abstract

Grape production is one of the most agronomically important activities worldwide. However, it is threatened by diseases caused by phytopathogenic microorganisms, which cause severe economic losses. The primary strategy to control phytopathogenic fungi is the application of fungicides; however, they affect the environment and induce resistance in fungi. Nanomaterials, especially those green-synthesized, emerge as an eco-friendly and sustainable alternative to control fungal pathogens. The objective of this work is to evaluate the sensitivity of fungal phytopathogens to biosynthesized copper-oxide nanoparticles (CuONPs). Nanoparticles were evaluated as preventive and corrective treatments in grapevine green tissues and fruits under field conditions, using in vitro and in vivo experimental approaches. Interestingly, corrective treatment was highly effective and showed little accumulation of Cu on the fruits, even less than a commercial copper-based fungicide. Moreover, we report that *Aspergillus niger* causes lesions in photosynthetic tissues and severe disease symptoms in grapes. We also describe for the first time the presence of *Alternaria alternata* causing lesions, mainly on the stems and young leaves of grapevine plants in Mexico. These pathogens were inhibited by the biosynthesized CuONPs. All these findings show the effectiveness of using CuONPs to control phytopathogenic fungi, even under field conditions, shedding light on their potential use in agriculture with a less environmental impact than the commercial fungicides and agrochemicals currently used.

## 1. Introduction

Grapes (*Vitis* genera, family Vitaceae) are cultivated in more than 7,450,000 hectares (ha) in various countries. This crop holds significant global economic value due to its contributions to both fruit production and the wine industry [1]. Mexico, ranking 11th in grape production worldwide, yields approximately 380,001 tons from around 37,000 ha, with the states of Sonora, Zacatecas, Baja California, Aguascalientes, Coahuila, and Querétaro being the top producers [2,3]. However, several phytopathogenic microorganisms, including bacteria, viruses, and fungi, pose severe threats to grapevines worldwide. Diseases such as powdery mildew, downy mildew, bacterial leaf spot, bunch rot, grapevine trunk diseases, vine canker, the grapevine red blotch virus (GRBV), and grapevine viruses A (GVA) and B (GVB) can have devastating effects on wine and grape production [4,5,6,7,8,9]. These pathogens attack different parts of the grapevine plants, including leaves, shoots, and even underground tissues, causing defoliation, reducing photosynthesis, impacting fruit quality, and causing significant economic losses. Unfortunately, controlling these diseases remains a major challenge. In this context, it is essential to explore alternatives for the prevention and treatment of these diseases, and bio-nanotechnology represents a promising option for addressing the issues associated with grapevine diseases.

Currently, the main alternative to control pathogenic fungi in vineyards and other commercially important crops is copper-based antimicrobial products (COBRETEC^®^, Cupehidro^®^, Cupravit^®^, Kocide^®^, Copper Green^®^, NORDOX^®^ 75W) and other agrochemical fungicides. However, the excessive use of these products in the soil can cause severe environmental pollution [10] and contribute to the development of resistance in phytopathogens against antimicrobial compounds [11,12]. Interestingly, the use of green-synthesized nanomaterials has been explored as a viable alternative to inhibit the growth of microbial pathogens, including fungi [13]. For instance, green-synthesized copper-oxide nanoparticles (CuONPs) have shown an excellent inhibitory effect against human pathogens such as *Candida albicans* [14] and *Campylobacter jejuni* [15], as well as other harmful bacteria [16]. Recent publications have documented the inhibitory activity of CuONPs against the phytopathogenic fungi *Alternaria alternata*, *Pyricularia oryzae*, *Botrytis cinerea*, and *Sclerotinia sclerotium* [17,18]. Furthermore, CuONPs clearly showed better antifungal activity compared with a commercial fungicide like Topsin-M 70 WP [18], drawing attention to their potential use for agricultural applications. Furthermore, green-synthesized nanomaterials are considered reliable, sustainable, and eco-friendly [19].

This study aimed to explore the effects of applying green-synthesized CuONPs on grapevine plants, searching for the feasibility of an alternative treatment for fungal infections. Then, for the first time, we analyzed the effect of green-synthesized CuONPs as a preventive and corrective treatment in grapevine plants in field conditions. CuONPs were applied twice a month as preventive treatment and weekly as corrective treatment in a vineyard from El Valle de Guadalupe, B.C., Mexico. From control plants showing lesions and not treated with CuONPs, we isolated *Aspergillus niger* and *A. alternata*, causing lesions in grapevine plants in Baja California, Mexico. Interestingly, these fungi were inhibited under in vitro conditions by CuONPs. All the findings suggest that CuONPs represent an alternative to control phytopathogens in the fields.

## 2. Materials and Methods

### 2.1. Synthesis of Nanoparticles

The synthesis of copper-oxide nanoparticles (CuONPs) was conducted as previously reported by [14], using a solution of copper sulfate pentahydrate (CuSO_4_·5H_2_O) (Sigma-Aldrich, St. Louis, MI, USA). Briefly, the CuSO_4_ solution was combined with the supernatant of *Trichoderma asperellum* (from the Microbiology Department collection), which served as a reducing agent. Sodium hydroxide (NaOH) was used to adjust the pH of the solution, which was then incubated at room temperature for 24 h. The formation of CuONPs was indicated by a noticeable change in the color of the reaction mixture. This process follows the reaction mechanism previously described by [20], and based on the stoichiometry of the reaction, the theoretical yield of CuONPs was calculated.

### 2.2. Characterization of Nanoparticles

The CuONPs were characterized using ultraviolet-visible spectrophotometry (UV-Vis), dynamic light scattering (DLS), and transmission electron microscopy (TEM). For UV-Vis characterization, a Perkin Elmer Precisely UV-VIS Lambda/25 spectrophotometer (PerkinElmer Inc., Waltham, MA, USA) was employed, with measurements taken in the wavelength range of 200 to 700 nm. The Zetasizer Nano ZS90 from Malvern (Malvern Panalytical Inc., Westborough, MA, USA) was used for DLS measurements to determine the hydrodynamic diameter and zeta potential (Z-potential) of the CuONPs. Finally, the morphology and size of the CuONPs were examined using a transmission electron microscope (TEM) (Hitachi H7500, Hitachi Ltd., Tokyo, Japan). To prepare the samples, 10 µL of CuONPs were placed on 75-mesh formvar/carbon-coated copper grids and allowed to dry at room temperature. The analysis was conducted at an accelerating voltage of 80 kV. The size of the nanoparticles was measured using the ImageJ V 1.54 software (free version for Windows 1.8.0_172).

### 2.3. Application of Nanoparticles in Grapevine Plants

Using a manual sprayer, copper-oxide nanoparticles (CuONPs) were applied to grapevine plants as both preventive and corrective treatment against phytopathogens in a vineyard located in the Valle de Guadalupe region, Baja California, Mexico. Ten Tempranillo (*Vitis vinifera* L.) plants were used for each treatment and control conditions. Four different concentrations of CuONPs were tested: 10% (*v*/*v*) (28.25 mg/L), 15% (*v*/*v*) (42.37 mg/L), 30% (*v*/*v*) (84.75 mg/L), and 45% (*v*/*v*) (127.12 mg/L). Preventive treatments were applied to the shoots, flowering buds, leaves, and fruits every two weeks over four months. The highest concentration (127.12 mg/L) was used as the corrective treatment, which was applied once a week for three weeks to plants exhibiting symptoms. The NORDOX^®^ 75W (84.4% Cu_2_O) fungicide was also used as a positive control of corrective treatments (at 1350 mg/L, according to the manufacturer). Plants were monitored for any signs of decay or any visible change in color.

### 2.4. Isolation of Fungi

Shoots, leaves, and fruits from the control plants, where the preventive treatment was not applied and which exhibited visible disease symptoms, were collected. Samples were transported under cool conditions to the laboratory. The plant tissues were rinsed first with tap water and then with sterile distilled water (SDW). Small pieces of the tissue, approximately 1 cm^2^ in size, were cut and placed on potato dextrose agar (PDA) medium containing ampicillin at 0.1 mg/mL. Petri dishes were incubated for 48 h at 25 ± 2 °C. Once fungal growth was observed, the fungal colonies were transferred to new Petri dishes until axenic cultures of the fungi were obtained. Both microscopic and macroscopic features of the isolated fungi were examined. DNA was extracted from the fungal isolates for molecular identification by sequencing the ITS regions, using the oligonucleotides ITS4 and ITS5 as described by [21].

### 2.5. Virulence Test

Virulence assays were conducted using Tempranillo shoot stems, berries, and leaves. The shoot stems of 10 cm and leaves were rinsed with tap water, washed once with 10% hypochlorite solution, and then rinsed with abundant sterile distilled water. Using a sterile scalpel, a 4 mm wound was made in each stem before inoculating them with a mycelial plug of 5 mm in diameter from either *A. alternata* or *A. niger*. Similarly, the grapes were washed consecutively with tap water and then 10% hypochlorite solution for 5 min, and then rinsed thoroughly with sterile distilled water before being inoculated with the fungal mycelial plugs. The experiments were incubated at room temperature under a 12 h light–dark cycle, and the symptoms were monitored daily. All assays were performed three times, each with three biological replicates.

### 2.6. In Vitro Antifungal Activity of Nanoparticles

The antifungal activity of nanoparticles was assessed against the fungal isolates *A. alternata* and *A. niger*. The fungal growth on poisoning media assays was performed using the same concentrations of CuONPs used in the field: 10% (28.25 mg/L), 15% (42.37 mg/L), 30% (84.75 mg/L), and 45% (127.12 mg/L). All assays were performed in triplicate, using the fungal growth on PDA media as a negative control. The diameter of the colonies was measured after 48 h at 25 °C and 30 °C for *A. alternata* and *A. niger*, respectively.

### 2.7. Determination of Cu in Plant Tissues Treated with CuONPs

At the end of treatment, samples of leaves and grapes for each treatment and controls were collected manually and dehydrated in an oven at 60 ± 2 °C until constant weight was obtained. The grapes and leaves were freeze-dried (Genesis Virtis 25ES, SP Scientific, PA, USA) for 3 days at −50 ± 2 °C and 0.01 mBar pressure. The dried samples were placed in plastic bags and kept in a cool, dark place until analysis. Copper determinations were made according to [22,23,24] as follows: To determine the concentration of copper deposited on the surface, approximately 0.5 g of the leaf sample and 1.5 g of the grapes were weighed. These samples were immersed in a 3% nitric acid solution (metal-free Fisher Scientific, Waltham, MA USA) for 10 min at room temperature (20 ± 2 °C). The solution was brought to a final volume of 10 mL in a volumetric flask and stored in plastic vials for later analysis.

To determine the total copper concentration, the grape and leaf samples were pulverized in a 30 mL agate mortar, and approximately 0.5 g of sample was weighed and placed in Teflon cups. The digestion of the samples was carried out by adding 15 mL of concentrated nitric acid, and they were covered with watch glasses to promote recirculation and avoid evaporation. The samples were heated on an iron at 250 ± 1 °C for 30 min, controlling the temperature with a PTC thermometer (573C Spot Check, CA, USA). Once the digestion was completed, the nitric acid was evaporated at a temperature below 100 ± 2 °C. The samples were brought to a final volume of 10 mL with 3% nitric acid and stored in plastic vials. During the process, reagent blanks were run as controls.

The analysis of the samples was carried out using VARIAN 220 FS atomic absorption equipment (AAS) with flame detection. The determination of the copper concentration was carried out at 324.8 nm. The air–acetylene mixture was used for analysis, and a copper cathode lamp was used for detection (Hollow Cathode Lamp Cu Varian, CA, USA). For calibration, Cu standard solutions were prepared (Sigma-Aldrich, St. Louis, MI, USA). An average of three absorbance readings was taken for all samples, including controls. The calibration curve was based on 4 concentrations (from 0 to 1.0 mg/L), obtaining a correlation coefficient of 0.999 and a detection limit of 0.0045 mg/L.

### 2.8. Processing of Plant Tissue for Transmission Electron Microscopy

Leaves exposed to CuONPs as a corrective treatment were processed for microscopic observation under transmission electron microscopy. Leaves without any treatment were used as the negative control, and as a positive control, leaves without any treatment were immersed in CuONPs (100%) for 24 h. With a sterile knife, the leaves were sectioned into small pieces of approximately 5 mm^2^. Then, they were fixed in a 2% glutaraldehyde solution for 30 min at room temperature. The samples were then washed with 1× PBS and post-fixed with 1% osmium tetroxide for 2 h at 4 ± 1 °C. Dehydration of the samples was performed using an increasing ethanol gradient: 25%, 50%, and 75% ethanol for 12 h, followed by 100% ethanol for 24 h. Following dehydration, the samples were infiltrated with a series of resin–ethanol mixtures: 25%, 50%, 75%, and 100% for 3 h each, and then, lastly, incubated overnight in 100% Spurr resin (Science Services, Copenhagen, Denmark). For flat embedding, the samples were placed on microscope slides with fresh 100% Spurr resin and polymerized at 60 ± 2 °C for 24 h. After polymerization, the samples were processed using a Leica Ultracut R ultramicrotome (Leica Microsystems Inc., Buffalo Grove, IL, USA). Sections of 70 nm were collected on 75-mesh copper grids coated with formvar/carbon and analyzed under a transmission electron microscope (Hitachi H7500, Hitachi Ltd., Tokyo, Japan) operated at 80 kV.

### 2.9. Statistical Analysis

The experiments were performed with the corresponding technical and biological replicates. The results are presented as means ± SD. Data were analyzed using GraphPad Prism 8.0.1. Significant differences among the mean values of different treatments were determined using Tukey’s test or Student’s *t*-test on a statistical analysis system (SAS). Graphs were created using GraphPad Prism.

## 3. Results

### 3.1. Nanoparticles Characterization

**Figure 1:**
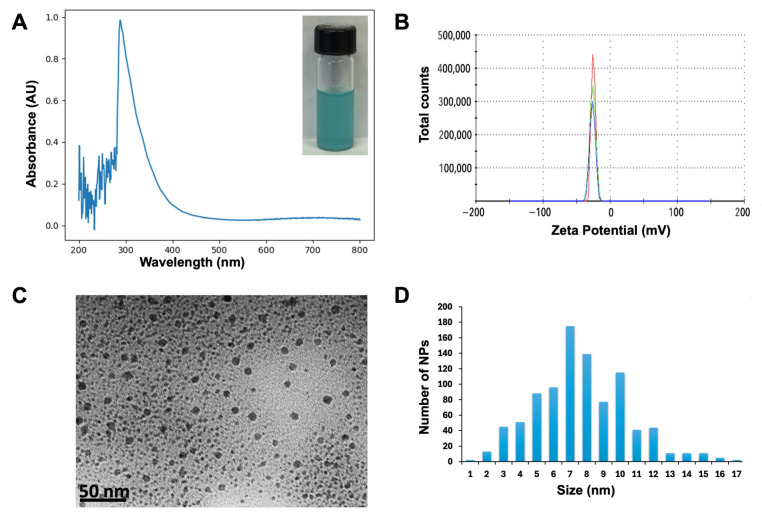
Characterization of the biosynthesized CuONPs. (**A**) Absorbance curve of CuONPs: inset shows freshly synthesized NPs. (**B**) Zeta potential of CuONPs: lines of different colors in the graph are the repetitions made when taking the measurement. (**C**) Transmission electron micrograph of CuONPs. (**D**) Size distribution histogram of CuONPs.

The formation of copper-oxide nanoparticles (CuONPs) was indicated by a color change to light blue in the solution (Figure 1A). UV-visible spectroscopy revealed that the synthesized nanoparticles exhibited a surface plasmon resonance (SPR) corresponding to nanometric copper, with a maximum peak at 280 nm, confirming the presence of CuONPs in the sample (Figure 1A). The hydrodynamic size and surface charge of the CuONPs were assessed using dynamic light scattering (DLS) analysis. The hydrodynamic size measured 143.3 nm, and the Zeta potential was −24.8 mV (Figure 1B). Transmission electron microscopy (TEM) analysis demonstrated that CuONPs are quasi-spherical (Figure 1C) and polydisperse, with size distributions spanning from 1 to 17 nm. A total of 1000 nanoparticles were measured, revealing a predominant population within this range. The particles exhibited an average diameter of 7.4 ± 2.8 nm, indicating significant heterogeneity in particle size (Figure 1C,D).

### 3.2. Plants Exposed to CuONPs as Preventive or Corrective Treatment Showed No Signs of Decay

**Figure 2:**
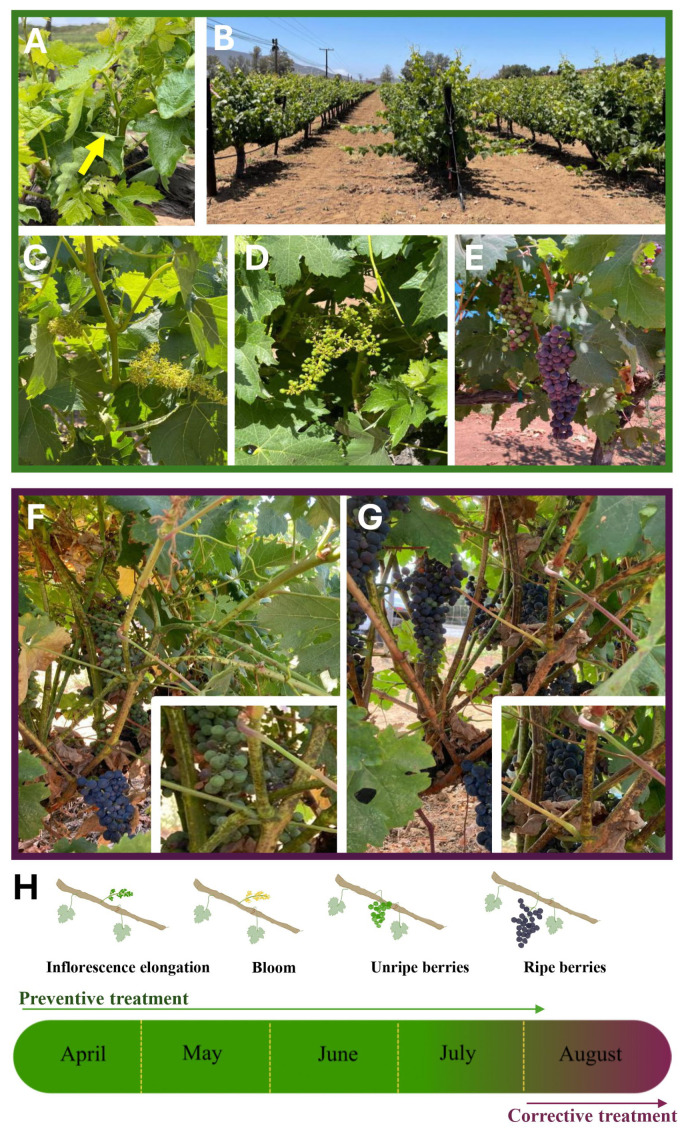
Plants exposed to CuONPs. (**A**) Preventive treatment started during the inflorescence formation stage. (**B**) Plants showed good fitness and normal development during the preventive treatment. (**C**–**E**) Plants showing normal development of blooming, unripe, and ripe berries during the preventive treatment. (**F**) Control plants without preventive treatment showed signs of fungal infection, and the corrective treatment was applied. Boxes in (**F**,**G**) show affected areas prior to and after corrective treatment, respectively. (**H**) Frame time of experimentation in the field. Months are highlighted in green and purple, when the preventive and corrective treatments were applied, respectively.

Plants treated with CuONPs were monitored once a week for visible signs of decay over leaves, stems, inflorescences, and unripe/ripe berries. Preventive treatment was applied to plants during the inflorescence formation stage (Figure 2A); over time, no signs of decay or change in color were evident in any of the treatments (Figure 2B). All plants grew with a normal and healthy appearance (Figure 2C,D). The flowering in treated plants developed similarly to that of control plants, as did the development of berries (Figure 2A,B). Grapes ripened normally, like the control plants without treatment (Figure 2C–E). Two control plants developed signs of fungal infection (Figure 2F,G); therefore, corrective treatment was applied weekly. Treatment was applied once a week for three weeks, directly over the signs of infection in fruits, leaves, and stems. After the corrective treatment, no difference was detected in the ripening of the grapes compared to those from control plants. As lesions were observed in the stems after the corrective treatment (inset of Figure 2F,G), small pieces were cut and transported to the laboratory under cold conditions. Stems were cut into 1 cm pieces and incubated over PDA at 25 °C. Interestingly, after a week of incubation, no fungal development was observed from the stems where corrective treatment was applied.

### 3.3. Plant Symptoms and Fungal Identification

**Figure 3:**
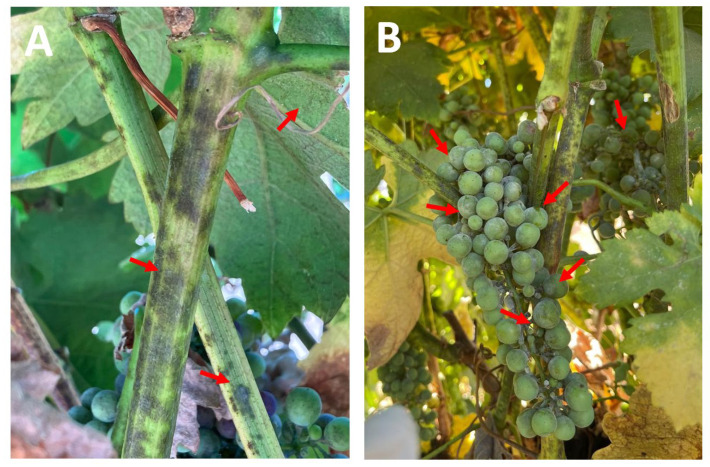
Representative pictures of the symptoms observed in vine plants. (**A**) Grayish-brown lesions were observed on the green leaves and stems (red arrows). (**B**) Early fungal infection in young grape berries were observed (red arrows).

**Figure 4:**
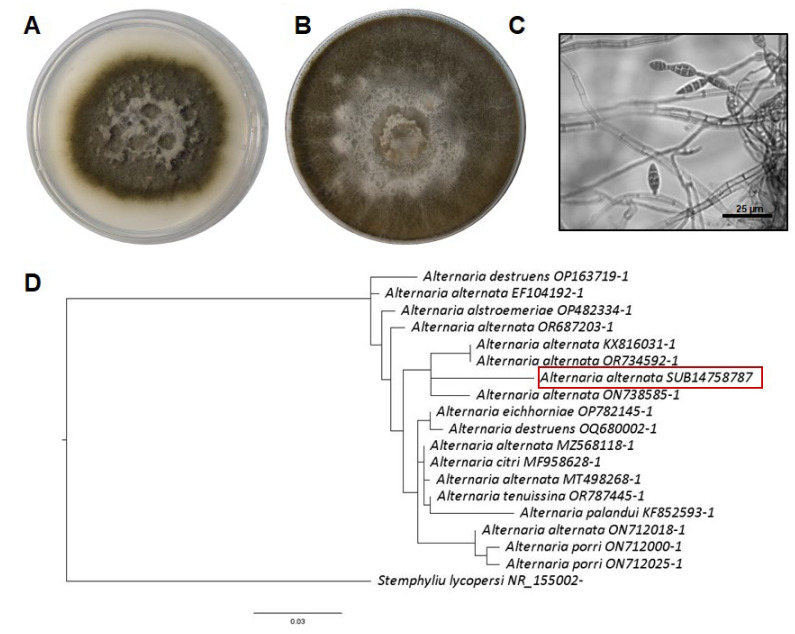
Identification of *A. alternata* isolate causing infection symptoms in grapevines. (**A**,**B**) Development of black fungal colonies on minimal and complete media, respectively. (**C**) Microscopic observation of the characteristic conidiophores of *A. alternata*. (**D**) Phylogenetic tree inferred from ITS sequences of selected species of *A. alternata*. The fungal isolate was grouped with the *A. alternata* species (red frame).

**Figure 5:**
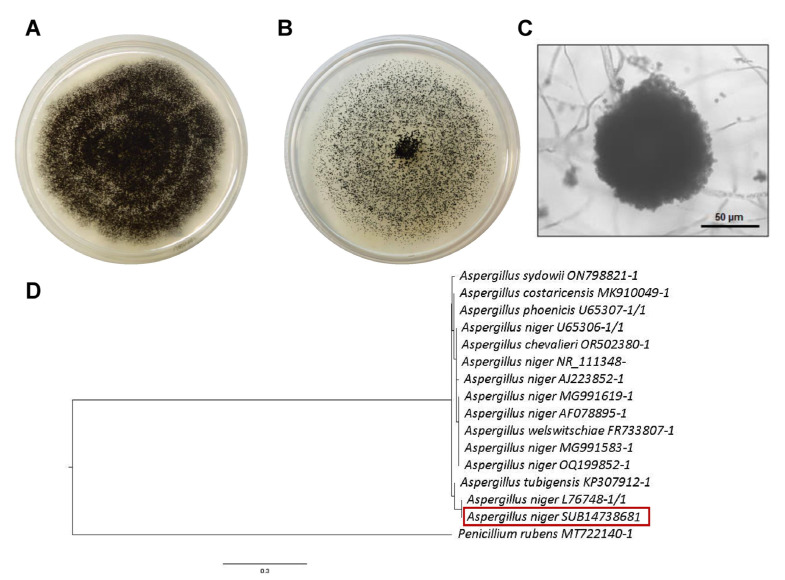
Identification of *A. niger* isolates causing infection symptoms in grapevines. (**A**,**B**) Development of black fungal colonies on minimal and complete media, respectively. (**C**) Microscopic observation of the conidiophores. (**D**) Phylogenetic tree inferred from ITS sequences of selected species of *A. niger*. The fungal isolate was grouped with the A. niger species (red frame).

Plants in the field, without any treatment, exhibited grayish-brown lesions on their green leaves and stems (Figure 3A). Additionally, unripe grapes showed symptoms of rotting (Figure 3B). One of the fungal isolates developed black colonies that grew rapidly in both minimal and rich media (Figure 4A,B). Under the microscope, septate hyphae and multicellular conidia were observed on short or elongated conidiophores (Figure 4C). These macro- and microscopic characteristics and the molecular identification confirmed that this fungus is *A. alternata* (Figure 4D). A second fungus was isolated from the plants with lesions, and on complete and minimal media, this fungus showed colonies that were initially white but that turned black during conidia production (Figure 5A,B). At the microscopic level, this fungus showed septate hyphae and radial black conidial heads (Figure 5C), which were morphological features of *A. niger*, which was then confirmed by sequencing (Figure 5D).

### 3.4. Virulence Analysis of Fungal Isolates

**Figure 6:**
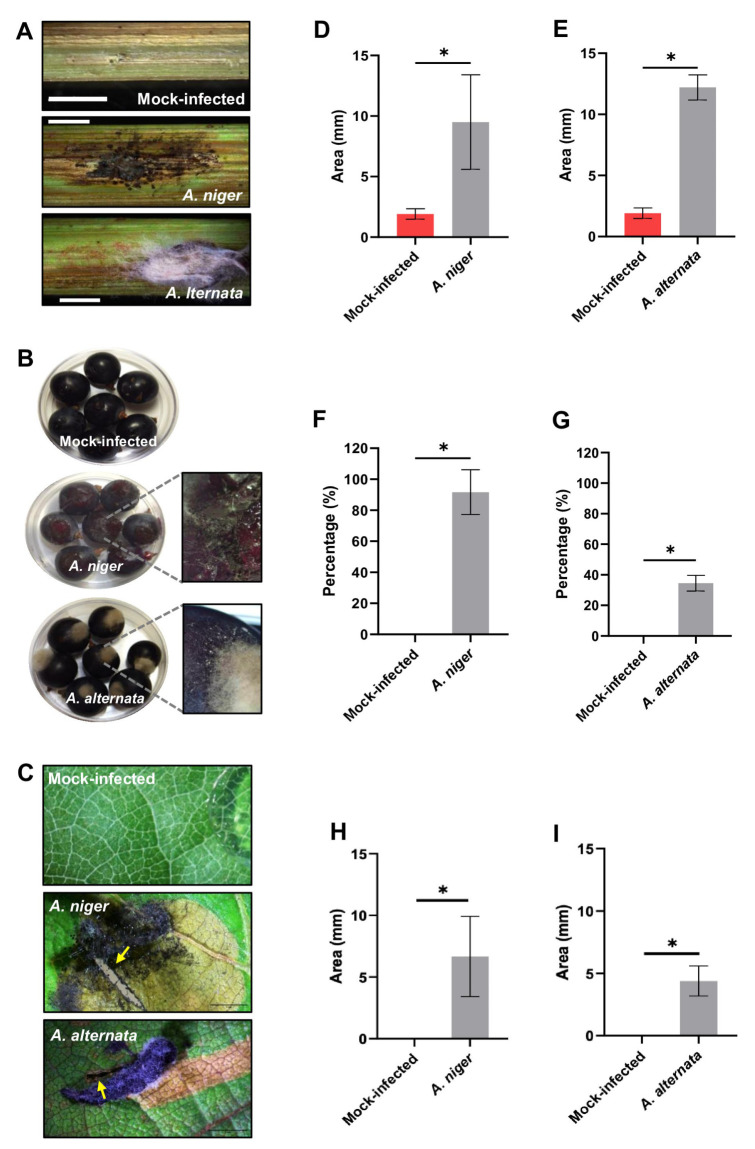
The *Alternaria alternata* and *Aspergillus niger* isolates cause rotting symptoms in grapevine plants. (**A**) Representative photographs of the lesions in stems. (**B**) Representative photographs of the rotting area on grape berries. (**C**) Representative photographs of fungi causing lesions in leaves. (**D**,**E**) Quantification of the lesion area on stems. (**F**,**G**) Percentage of grape berries showing rooting symptoms. (**H**,**I**) Measurement of the damaged area on leaves. Arrows indicate wounds made in green tissues. Scale bars = 2 mm in (A) and 1 mm in (C). Asterisks in the bar graphs indicate significant differences determined by a Student’s *t*-test, where * *p* < 0.05.

Healthy stems, leaves, and grapes were inoculated with isolates identified as *A. niger* and *A. alternata* (Figure 6). Mock-infected stems exhibited small lesions corresponding to the inoculation site, and the mock-infected grapes and leaves did not show lesions or infection symptoms (Figure 6A–C). The stems inoculated with the *A. niger* exhibited lesions measuring 9.4 mm in length (Figure 6A,D), all the inoculated grapes exhibited severe symptoms of rot (Figure 6B,F), and leaves inoculated with this fungus showed lesions of 6.6 mm (Figure 6C,H). Notably, stems infected with *A. alternata* displayed lesions measuring 12.2 mm in length (Figure 6A,E). Additionally, 34% of the inoculated grapes showed growth of *A. alternata* on the inoculated grapes (Figure 6B,G), and this fungus caused lesions of 4.3 mm in leaves (Figure 6C,I).

### 3.5. CuONPs Showed Antifungal Activity In Vitro

**Figure 7:**
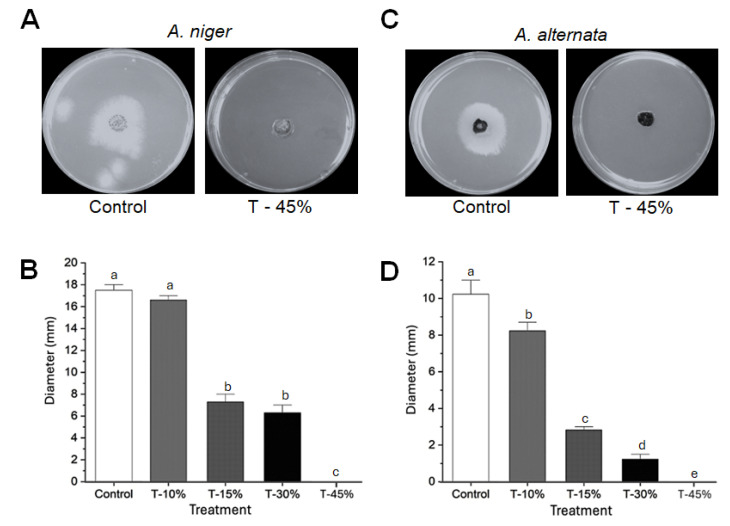
Inhibition in vitro of fungal isolates by CuONPs. (**A**,**C**) Representative photographs of the inhibition growth of *A. niger* and *A. alternata* in PDA added with CuONPs. (**B**) Quantification of the inhibition of *A. niger*. (**D**) Quantification of the inhibition of *A. alternata*. Different letters in B and D denote significant differences (analysis of variance, Tukey).

Poisoning assays revealed that *A. niger* showed lower susceptibility to CuONPs. However, fungal growth was significantly reduced on media supplemented with 15% and 30% CuONPs (Figure 7A,B). For *A. niger,* colony diameter in the control plate was 17.5 ± 0.4 mm, while at 15% of CuONPs, this was reduced to 7.2 ± 0.8 mm, and at 30%, 6.4 ± 0.7 mm. In contrast, *A. alternata* was highly susceptible to all tested concentrations of CuONPs (Figure 7C,D). At the highest concentration tested (45%), corresponding to 127.12 mg/L, both fungi were susceptible, and no further growth was recorded (Figure 7A,C). Colony diameter in the control plate was 10.2 ± 0.9 mm, while at 15% of CuONPs, it was reduced to 2.8 ± 0.2 mm, and at 30%, 1.3 ± 0.3 mm.

### 3.6. Plants Treated with CuONPs Showed Low Concentration of Cu in Tissues

**Figure 8:**
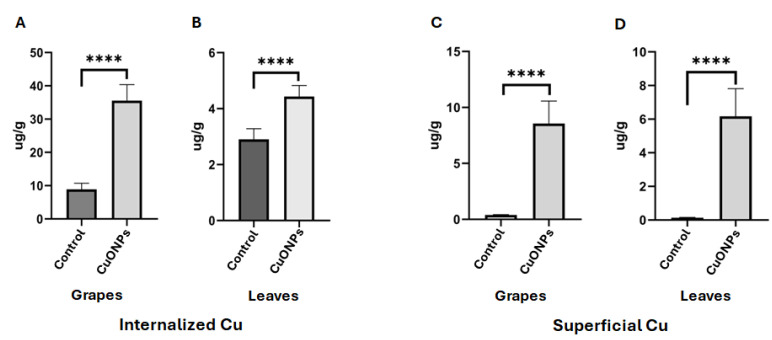
Quantification of copper accumulated in plant tissues after the preventive treatment with CuONPs. (**A**,**B**) Quantification of copper in grapes and leaves, respectively. (**C**,**D**) Quantification of copper deposited on the surface of grapes and leaves, respectively. Asterisks in the bar graphs indicate significant differences determined by a Student’s *t*-test for each independent plant tissue treated with CuONPs. Asterisks indicate significant differences.

To evaluate the concentration of Cu accumulated in the tissues of plants treated with nanoparticles as a preventive treatment, the levels of Cu in grapes and leaves were measured (Figure 8). The average concentrations found were 35.5 μg/g in the treated grapes and 5.4 μg/g in the treated leaves, both of which surpassed the levels of Cu found in untreated plant tissues (Figure 8A,B). Taking into consideration that some Cu may remain on the surfaces of the leaves and grapes, we proceeded to analyze the concentration of Cu on the surface of tissues. The results indicated a Cu concentration of 8.5 μg/g on the surface of grapes and 6.1 μg/g on the surface of leaves (Figure 8C,D). Control plants not treated with nanoparticles exhibited Cu concentrations close to zero on their surfaces (Figure 8).

**Figure 9:**
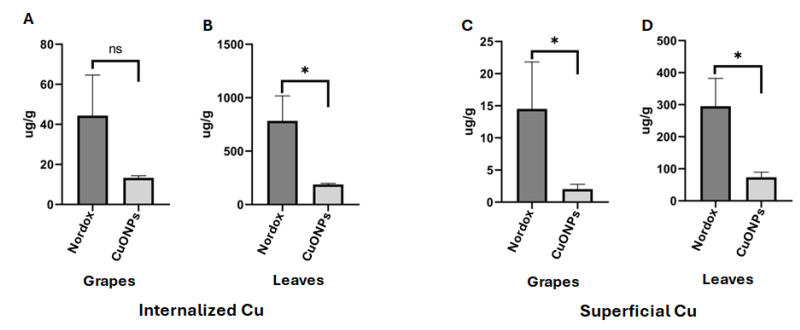
Quantification of the copper accumulation in plant tissues after the corrective treatment with CuONPs or NORDOX^®^ 75W fungicide. (**A**,**B**) Quantification of copper in grapes and leaves, respectively. (**C**,**D**) Quantification of copper deposited on the surface of grapes and leaves, respectively. Notice a higher quantification of Cu in plant tissues treated with the fungicide NORDOX^®^ 75W. Asterisks in the bar graphs indicate significant differences determined by a Student’s *t*-test, where * *p* for each independent plant tissue treated with CuONPs or NORDOX^®^. "ns” indicates non-significant differences.

Additionally, we employed CuONPs as a corrective treatment for grapevines exhibiting symptoms of infection by *A. alternata* and *A. niger*. After the nanoparticle application period, the concentrations of copper were quantified in both homogenized plant tissues and the copper deposited only on the surface of tissues (Figure 9). For reference, the fungicide NORDOX^®^ 75W containing cuprous oxide as an active compound was used as a positive control. Notably, 13.3 μg/g of copper in the grapes and 188.5 μg/g in the leaves were found in those plants treated with CuONPs (Figure 9A,B). The copper on the surface of tissues of these CuONP-treated plants showed 2.0 μg/g of copper for grapes and 73.3 μg/g for leaves (Figure 9C,D). In contrast, the concentrations of copper in grapes and leaves from NORDOX^®^ 75W-treated plants were considerably higher, measuring 44.3 μg/g and 782.5 μg/g, respectively (Figure 9A,B). The copper on the surface of tissues from grapes and leaves treated with NORDOX^®^ 75W displayed a copper concentration of 14.5 μg/g and 295.0 μg/g, respectively (Figure 9C,D).

### 3.7. Leaves Exposed to CuONPs Maintain Cell Shape, but Excessive Exposure Provokes Apparent Metallic Accumulation in Vacuoles

**Figure 10:**
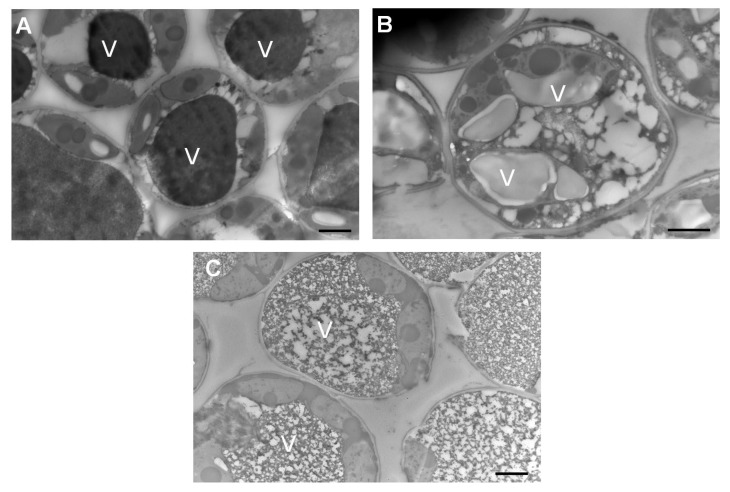
Transmission electron micrographs of leaf tissue from field-grown grapevines. (**A**) Cells of leaf taken from the field and exposed to CuONPs for 24 h. (**B**) Cells of leaf exposed to CuONPs as a preventive treatment. (**C**) Cells of leaf without nanoparticle treatment; scale bars = 2 µm. V = vacuole.

Leaves that received preventive treatment with CuONPs were processed and examined using transmission electron microscopy. Leaves without any treatment were used as a control, and leaves immersed in CuONPs for 24 h were used as a positive control. To detect any metallic accumulation, samples were not post-stained. Plant cells that were immersed in CuONPs as a positive control exhibit a dense accumulation in vacuoles (Figure 10A). This contrasted with plant cells that received CuONPs as a preventive treatment, which did not exhibit dense accumulations in vacuoles (Figure 10B). As expected, plant cells that were not treated with CuONPs did not show any dense accumulation in their vacuoles (Figure 10C).

## 4. Discussion

Grape production and the wine industry are important agro-industrial activities worldwide. In Mexico, table grapes, wine, and raisins marketing represented approximately USD 45 million in 2022 [25]. However, grape production is severely affected by the presence of phytopathogens such as viruses [26], bacteria [27], oomycetes [28], and fungi [7,26]. Most of the research about fungal pathogens of grapes is based on the study of those fungi affecting blooms, ripe fruit, fruits at post-harvest stages [29,30], and wood fungi [6,7], for instance, the fungus *Botrytis cinerea*, causal agent of the gray mold or bunch rot [29], or fungi from the Botryosphaeriaceae family, including species of *Botryosphaeria*, *Lasiodiplodia*, *Neofusicoccum*, and *Diplodia* [7].

Searching for frontier alternatives for controlling fungi infecting photosynthetic tissues such as young stems, leaves, and unripe fruits in grapevine plants, we synthesized CuONPs using the supernatant of *T. asperellum* as a reducing agent. These nanoparticles were tested in a previous study, showing excellent inhibition of *Candida albicans*, a human pathogen [14]. In this work, for the first time, CuONPs in a vineyard were tested as a preventive treatment every 15 days at different concentrations, from the inflorescence elongation stage to the ripening of grape berries. Plants were kept under observation, and no apparent decay or damage was detected (Figure 2B). Also, CuONPs were used as corrective treatment in the vineyard, applying them once a week for three weeks. After these applications, we evaluated the amount of copper in the fruits and leaves at the highest concentration tested (45%), corresponding to 127.12 mg/L. In the preventive treatment, the grape berries accumulated a greater quantity of copper (35.5 μg/g) than the leaves (4.42 μg/g), but the copper deposited on the surface of leaves and fruit was comparable (6.8 and 8.5 μg/g, respectively). The higher accumulation of copper in grapes after the preventive treatment is not surprising, since the applications were for an extended period, starting from the inflorescence elongation stage to ripening. Although the number of stomata is variable between grape cultivars [31,32], it is possible that CuONPs may enter through the stomata and lenticels of grape berries, which are present before bloom and until harvest [32].

As a corrective treatment for symptomatic plants, the commercial fungicide NORDOX^®^ 75W was applied in the field, to compare the accumulation of copper by applying CuONPs and a copper-based commercial product. Interestingly, those plants treated with NORDOX^®^ 75W showed higher accumulations of copper (782.5 μg/g in leaves and 44.3 μg/g in grapes) compared to those treated with CuONPs (188.5 μg/g and 13.3 μg/g for leaves and grapes, respectively). The higher accumulation of copper in leaves using both treatments could be due to lower metabolic activity and decreased nutrient translocation from mature leaves to other plant tissues during the corrective treatment [33]. It is possible that copper accumulates in vacuoles after prolonged exposure to CuONPs, as suggested by transmission electron microscopy analysis, revealing a dense accumulation in vacuoles from leaves immersed for 24 h in the CuONPs suspension, compared to leaves exposed to the preventive treatment (Figure 10). Foliar uptake of nanoparticles has been reported by [34]; they applied TiO_2_NPs for three weeks on grapevine leaves (*Vitis vinifera* L.) under field conditions. They did not analyze the fruits, but leaf-to-leaf translocation was not detected. Little is known about how grapevine leaf cells respond to metallic NPs. However, it was evident that cells not exposed to CuONPs had typical, large vacuoles filled with protein deposits [35] (Figure 10C). In contrast, vacuoles of cells exposed to CuONPs showed altered morphology, which was more evident in cells exposed directly to the undiluted suspension of CuONPs for 24 h.

Although we found a higher accumulation of copper in the grape berries in the preventive treatment, the concentration of the metal was lower in grapes exposed to the corrective treatment (13.3 μg/g), a value not far from that recorded for the control grapes without any treatment (8.91 μg/g). From the results using CuONPs as corrective treatment in grapes, we can assume that these copper concentrations will not significantly affect the winemaking process. It has been well-documented that copper decreases considerably during winemaking [36,37]. Thus, modern nanotechnology is promising for a wide range of applications in viticulture and enology [38,39]. In fact, several nanomaterials have been proposed as nanofertilizers to improve grape yield and quality, thus reducing toxic agrochemicals [40,41]; to control microbes in vineyards and wine production; to improve wine analysis techniques; and for their use in innovative packaging, among other applications [39].

Recently, green-synthesized silver nanoparticles were evaluated on grapevine plants, and a biostimulant effect was determined, based on the number of grapes produced and on the length of the young shoots [42]. Similarly, shoot length and yield per grapevine were enhanced, and the berries’ quality improved by applying silica nanoparticles (SiNPs) on Thompson Seedless grapevines. Furthermore, grapevines infected with downy mildew were evaluated, and a reduction of 81.5% in disease severity was found. However, they found cytotoxic and genotoxic effects at the doses tested, suggesting further investigations to determine the SiNP residue in the produced edible plant parts [43]. The cytotoxic effects of nanomaterials are dose-dependent, and other factors like size and shape are also critical. The CuONPs used in this study were previously evaluated in different mammalian cell lines, and the results showed good biocompatibility and hemocompatibility at the concentrations tested [14]. Therefore, our results comparing the concentration of copper in the edible parts of the grapevines, when using CuONPs and a copper-based commercial product, suggest that CuONPs analyzed in this work could be safely applied as an antifungal agent in a corrective manner.

On the other hand, according to our knowledge, this is the first report of *A. alternata* causing lesions, mainly in young stems and leaves of grape plants in Mexico. The young vine tissues showed grayish-brown lesions on the leaves and stems, and rot in the unripe fruits. However, *A. alternata* has previously been reported to cause light symptoms of infection in grapevine plants and release toxins affecting the fruit quality [44]. Interestingly, *A. niger* infection of grape plants has been previously reported as a grapevine trunk disease [7]. In this work, we report on *A. niger* affecting photosynthetic tissues and fruits of grapevine plants, and severe fruit rot was observed in grapes inoculated with *A. niger* (Figure 6B) compared to mock-infected ones. Infection assays confirmed that these symptoms and lesions on young photosynthetic tissues and fruits were caused by the fungal isolates.

Finally, we report on the effective use of CuONPs to control these pathogens in the field. Interestingly, the growth of *A. niger* isolates was inhibited by 10%, 15%, and 30% concentrations of nanoparticles under in vitro conditions. Similarly, *A. alternata* isolates showed inhibition at concentrations of 10% of nanoparticles in the culture medium, suggesting that the suspension of CuONPs could be used at lower concentrations in mild infections, significantly reducing the amount of copper accumulated in grapes.

## 5. Conclusions

The use of green-synthesized CuONPs was attempted for the first time in the field on grapevine plants, as preventive and corrective treatment against phytopathogenic fungi. Both treatments effectively controlled the plant colonization by fungi. The plants did not present any decay or damage at any of the concentrations tested (10, 15, 30, and 45% of CuONPs), and the fruits showed normal development and ripening. From the control plants showing lesions and which were not treated with CuONPs, we isolated *A. niger*, and for the first time, we report *A. alternata* as a responsible agent for lesions in the shoots and leaves of grapevine plants in Baja California, Mexico. The fungal isolates, *A. niger* and *A. alternata*, were susceptible to CuONPs under both in vivo and in vitro conditions. The accumulation of copper in grapes subjected to preventive and corrective treatments suggests that CuONPs may be used as a corrective treatment with reduced copper accumulation in the grapes. Collectively, these findings open the possibility of reducing the use of toxic agrochemicals in the field and demonstrate that using CuONPs significantly reduces the amount of accumulated copper in grapes compared with a commercial copper-based fungicide. Overall, the findings presented in this work suggest that CuONPs could be used for corrective treatments, representing a viable alternative for controlling phytopathogens in fields of cultivation.

## Data Availability

Data are contained within the article.
